# Case Report: Severe hypernatremia from psychogenic adipsia

**DOI:** 10.12688/f1000research.9181.1

**Published:** 2017-01-11

**Authors:** Sarah Manning, Rehan Shaffie, Shitij Arora

**Affiliations:** 1 Division of Internal Medicine, Montefiore Medical Center, Albert Einstein College of Medicine, Bronx, USA

**Keywords:** hypernatremia, adipsia, osmolality, altered mental status, osmotic demyelination, paranoia, stroke, infection

## Abstract

Hypernatremia is a common emergency room presentation and carries high mortality. We describe a case of a 56-year-old male patient with who presents with refusal to drink water for several weeks leading to the admission. He was diagnosed with psychogenic adipsia and was treated successfully with fluids, mirtazapine and clonazepam.

## Introduction

Hypernatremia is a common electrolyte abnormality seen in the emergency department and can carry an estimated mortality of 40–60% depending on the degree of severity ^1^. Psychogenic adipsia is a rare cause of hypernatremia and represents a subgroup where chronic long term management is critical as these patients are likely to relapse. There has been a reported case where hypernatremia has been corrected with hemodialysis using a high sodium dialysate to prevent osmotic demyelination syndrome ^2, 3^. One of the mechanisms involved in psychogenic adipsia is the destruction or improper functioning of osmoreceptors in the hypothalamus that controls the thirst mechanism; this may be a result of a congenital malformation or acquired as in the case of stroke, trauma, or infection ^4, 5^.

We report a case of hypernatremia with serum sodium as high as 181meq/l and no neurologic manifestations, after patient refused to drink water in the nursing home.

## Case presentation

The patient being described is a 56 year old male with cognitive developmental delay and anxiety who is sent from his assisted living facility with hypernatremia on routine labs and documented refusal to drink water. There were no other complaints. He was afebrile and recorded a blood pressure of 99/97mmHg. Physical exam showed a cachectic male with dry mucous membranes. A complete medication list provided by the patient’s assisted living facility included famotidine 40 mg daily, docusate 100 mg daily, and a daily multivitamin.

Laboratory analysis showed a plasma sodium concentration of 181 mEq/L, plasma chloride concentration of 138 mEq/L, and plasma potassium concentration of 4.6 mEq/L. Serum osmolality was revealed to be 359 mOsm/kg. The urine sodium level was less than 20 mEq/L and the urine chloride level was also less than 20 mEq/L. Urine osmolality was 1080 mEq/L. The patient was immediately rehydrated with D5 1/2 normal saline solution not to exceed a correction rate of 6–8 mEq/L of sodium per day. The patient continued to refuse most oral intake and denied thirst. A CT scan was obtained without contrast and showed mild microvascular ischemic disease without evidence of intraparenchymal hemorrhage, acute infarct, or hydrocephalus. No hypothalamic infarct or other mass lesion or focal mass effect were seen. Later in the course of his admission he admitted to severe stress from a recent emotional break up. He was started on mirtazapine 7.5 mg daily and clonazepam 0.25 mg twice daily to address his anxiety and that led to an improvement in appetite and regained thirst mechanism. He was stable when discharged back to his assisted living facility. His sodium remained stable within the normal range when discharged back to his assisted living facility and was normal at 6 months post discharge follow up.

## Discussion

The above case describes a patient with profound hypernatremia devoid of thirst and remarkably asymptomatic on neurologic exam. There are at least two very interesting phenomenon that can be discussed through this case. One is the presence of an intense emotional response and its effect on the thirst mechanism. Thirst is a very powerful mechanism meant to protect against hypernatremia. Functional MRI studies have demonstrated anterior cingulate gyrus as the core are associated with the consciousness of thirst ^6^. The same area is also implicated in a number of psychiatric disorders like schizophrenia depression and autism ^7^. While osmoreceptors sense the plasma sodium levels, the consciousness of thirst involves a very different and complex limbic system involvement. A patient with a plasma sodium concentration of 150 mEq/L or more who is alert but not thirsty has, by definition, a hypothalamic lesion affecting the thirst center ^8^. Psychiatric illness affecting osmoreceptors of the hypothalamus appears to be very rare and very few cases have been reported; one of them involved a 17-year-old boy with psychosis who displayed an impaired thirst mechanism similar to the patient described above. When the psychosis was treated and began to resolve, the thirst mechanism returned ^9^. A detailed psychiatric history should be very useful in preventing recurrences and identifying cases with psychogenic adipsia.

The case also highlights the cerebral adaptation to chronic hypernatremia that results in absent neurologic sequelae. The latter response involves an initial uptake of sodium and potassium, followed by the later accumulation of osmolytes; mainly myo-inositol and the amino acid glutamine. The delayed efflux of these osmolytes as seen when the sodium is corrected too rapidly, is what results in cerebral edema, seizures and coma ^10^. 

In conclusion, psychogenic adipsia represents a rare cause of severe hypernatremia and this case highlights the importance of psychiatric history in patients who present with severe chronic or recurrent hypernatremia.

## Consent

Written informed consent for publication of the patient’s details was obtained from the patient.
