# Supplementary material for: Case Report: Severe hypernatremia from psychogenic adipsia
Source: F1000Res. 2017 Jan 11;6:34. [Version 1] doi: 10.12688/f1000research.9181.1 (PMC5265699; doi:10.12688/f1000research.9181.1)
Supplement: Peer review history

# Peer review: Referee response for version 1

**DOI:** 10.5256/f1000research.9879.r19222

**Published:** 2017-01-20

**Authors:** Bijin Thajudeen

**Affiliations:** 1 Division of Nephrology, Banner - University Medical Center Tucson (BUMCT), University of Arizona , Tucson, AZ, USA

Interesting case. It would be more interesting if authors can comment of the role played by mirtazapine in curing the adipsia. One of the side effects of mirtazapine is increased thirst. Mirtazapine increases dopaminergic neurotransmission and dopamine has role in the modulation of thirst. On reviewing some of the case reports which deals with adipsia and psychiatric disorders (depression, schizophrenia), the treatments or intervention (electroconvulsive therapy) used have one thing in common. They all increase dopaminergic activity, supporting the hypothesis that deficiency of dopamine or lack of dopaminergic activity may be playing role in the pathogenesis of adipsia.

 Adipsic hyponatremia is uncommon in patients with psychiatric disorders. Hence secondary causes like tumour, histiocytosis, sarcoidosis involving the hypothalamus should be ruled out. An MRI of the brain with or without contrast would be the most appropriate investigation of choice rather than a CT head without contrast. 

 Patients with adipsic hyponatremias associated with psychiatric disorders will have a normal ADH response and an appropriate increase in urine osmolality.

I have read this submission. I believe that I have an appropriate level of expertise to confirm that it is of an acceptable scientific standard.

## References

[1] MittlemanGRosnerALSchaubCL: Polydipsia and dopamine: behavioral effects of dopamine D1 and D2 receptor agonists and antagonists. *J Pharmacol Exp Ther*.1994;271(2) :638-50 7965779

[2] NakayamaKSakuraiTKatsuH: Mirtazapine increases dopamine release in prefrontal cortex by 5-HT1A receptor activation. *Brain Res Bull*.2004;63(3) : 10.1016/j.brainresbull.2004.02.007 237-41 10.1016/j.brainresbull.2004.02.007 15145142

[3] SakerPFarrellMJAdibFREganGFMcKinleyMJDentonDA: Regional brain responses associated with drinking water during thirst and after its satiation. *Proc Natl Acad Sci U S A*.2014;111(14) : 10.1073/pnas.1403382111 5379-84 10.1073/pnas.1403382111 24706817PMC3986125

[4] PhillipsMGGabowPA: Psychogenic adipsia in a patient with psychotic depression. *Am J Kidney Dis*.1990;15(6) :592-4 236869810.1016/s0272-6386(12)80531-2

[5] LandauAMChakravartyMMClarkCMZisAPDoudetDJ: Electroconvulsive therapy alters dopamine signaling in the striatum of non-human primates. *Neuropsychopharmacology*.2011;36(2) : 10.1038/npp.2010.182 511-8 10.1038/npp.2010.182 20944554PMC3055667

[6] FarleyPCLauKYSubaS: Severe hypernatremia in a patient with psychiatric illness. *Arch Intern Med*.1986;146(6) :1214-5 3718109

# Peer review: Referee response for version 1

**DOI:** 10.5256/f1000research.9879.r19526

**Published:** 2017-01-20

**Authors:** Amarpali Brar

**Affiliations:** 1 Department of Medicine, SUNY Downstate Medical Center, Brooklyn, NY, USA

Sarah Manning and co-authors present a case of psychogenic adipsia and hypernatremia. Overall the case report is well written. Although previously described as cited by authors and also additionally reported by others as listed below ^1^^,^^2^, this is a known clinical presentation.

 This case report will add to literature on this rare clinical presentation.

 Urine osmolality units should be changed to mOsm/kg.

 Add other published reports about this presentation in discussion.

I have read this submission. I believe that I have an appropriate level of expertise to confirm that it is of an acceptable scientific standard.

## References

[1] PhillipsMGGabowPA: Psychogenic adipsia in a patient with psychotic depression. *Am J Kidney Dis*.1990;15(6) :592-4 236869810.1016/s0272-6386(12)80531-2

[2] ChoiJHLeeHSKimSMKimHYKwonSK: Paranoid Adipsia-induced Severe Hypernatremia and Uremia treated with Hemodialysis. *Electrolyte Blood Press*.2013;11(1) : 10.5049/EBP.2013.11.1.29 29-32 10.5049/EBP.2013.11.1.29 23946763PMC3741440
